# UK Medical Cannabis Registry: a case series analyzing clinical outcomes of medical cannabis therapy for generalized anxiety disorder patients

**DOI:** 10.1097/YIC.0000000000000536

**Published:** 2024-02-02

**Authors:** Adam Li, Simon Erridge, Carl Holvey, Ross Coomber, Daniela Barros, Urmila Bhoskar, Matthieu Crews, Lorna Donnelly, Muhammad Imran, Laura Korb, Gracia Mwimba, Simmi Sachdeva-Mohan, James J. Rucker, Mikael H. Sodergren

**Affiliations:** a Medical Cannabis Research Group, Department of Surgery and Cancer, Imperial College London; b Sapphire Medical Clinics; c St. George’s Hospital NHS Trust; d North London Mental Health Partnership; e Department of Psychological Medicine, Kings College London; f South London & Maudsley NHS Foundation Trust, London, UK

**Keywords:** anxiety, cannabidiol, cannabinoids, cannabis, tetrahydrocannabinol

## Abstract

This study aims to analyze changes in health-related quality of life (HRQoL) and safety in patients with generalized anxiety disorder (GAD) prescribed a homogenous selection of cannabis-based medicinal products (CBMPs). Patients prescribed Adven CBMPs (Curaleaf International, UK) for GAD were identified from the UK Medical Cannabis Registry. Primary outcomes were changes in patient-reported outcome measures (PROMs) from baseline up to 12 months, including GAD-7, Single-Item Sleep Quality Scale (SQS), and EQ-5D-5L. Adverse events were recorded using CTCAE version 4.0. A total of 120 patients were identified for inclusion, of which 38 (31.67%), 52 (43.33%), and 30 (25.00%) were prescribed oils, dried flower, and both formulations of CBMP. Associated improvements in GAD-7, SQS, and EQ-5D-5L at 1, 3, 6, and 12 months were observed compared to baseline (*P* < 0.010). There were 24 (20.00%) patients who reported 442 (368.33%) adverse events, most of which were mild (n = 184, 41.63%) and moderate (n = 197, 44.57%). This study reports an association between initiation of a homogeneous CBMP therapy and improvements in anxiety severity and HRQoL in individuals with GAD. Moreover, therapy was well-tolerated at 12 months follow-up. Further investigation through randomized controlled trials will ultimately be required to determine causation.

## Background

Generalized anxiety disorder (GAD) is defined as disproportionate, persistent, and excessive anxiety for a minimum of 6 months (DeMartini *et al*., 2019). The prevalence of GAD in the adult English population is estimated to be 5.9% (NHS, 2014). GAD patients have an increased risk of impairment in mental health, social functioning, and overall well-being (Comer *et al*., 2011). GAD is consequently the most impairing anxiety disorder (Comer *et al*., 2011). With anxiety disorders estimated to have an annual direct cost of $42.3 billion globally (Remes *et al*., 2018), the health and economic burden of GAD on patients and wider society is evident.

Despite the pharmacotherapeutic options available, only 60–85% of patients with anxiety disorders experience at least a 50% improvement in symptoms (Garakani *et al*., 2020). Moreover, a study by the Harvard/Brown Anxiety Disorders Research Program reported over a 12-year follow-up period, there was a 0.45 probability of GAD recurrence in patients who had previously recovered (Bruce *et al*., 2005), demonstrating a need for novel therapeutic options.

Cannabis-based medicinal products (CBMPs) are derived from cannabis, of which (−)-trans-Δ^9^-tetrahydrocannabinol (THC) and cannabidiol (CBD) are the major active pharmaceutical ingredients (Mechoulam, 2005). THC is a partial agonist of cannabinoid type 1 (CB1) receptors (Dutta *et al*., 2022). THC has demonstrated conflicting effects with respect to anxiety. Stimulation of CB1 receptors in the prefrontal cortex and ventral hippocampus of rats has been associated with anxiolytic effects (Rubino *et al*., 2008). However, stimulation in the basolateral amygdala results in anxiogenic behavior, even at low doses (Rubino *et al*., 2008). It has since been established in pre-clinical studies that CB1 agonists have a biphasic response on anxiety, whereby they are anxiolytic at low doses, but anxiogenic at high doses (Rey *et al*., 2012; Stoner, 2017). A 2020 review of pre-clinical and clinical studies on CBD treatment for anxiety stated that the pharmacology of CBD is not fully understood (Wright *et al*., 2020). However, CBD has been shown to have anxiolytic effects by interacting with serotonin 5-HT1A receptors, cannabinoid type 1 and 2 receptors, and transient receptor potential vanilloid 1 channels in the central and peripheral nervous system (Wright *et al*., 2020).

There have been two randomized controlled trials which have examined the outcomes in individuals prescribed CBD for social anxiety disorder (Bergamaschi *et al*., 2011; Crippa *et al*., 2011; Black *et al*., 2019). Participants were prescribed between 400-600 mg of CBD prior to anxiety-inducing event, with each study finding a benefit in reported outcomes (Bergamaschi *et al*., 2011; Crippa *et al*., 2011). This was not maintained on pooling of outcomes (Black *et al*., 2019). A systematic review investigating changes in anxiety with pharmaceutical THC treatment with or without CBD reported an associated improvement in anxiety-specific outcomes (Black *et al*., 2019). However, this data is obtained from studies where participants were primarily prescribed CBMPs for indications other than anxiety disorders, and the evidence quality was described as very low (Black *et al*., 2019). This may be attributed to the heterogeneity of study methodologies, with inconsistent administration and formulations of CBMPs (Häuser *et al*., 2018; Montero-Oleas *et al*., 2020). This results in inconsistent cannabinoid concentrations and makes drawing conclusions on CBMP effectiveness in the context of anxiety and GAD difficult (Banerjee *et al*., 2022). Moreover, existing studies frequently use isolate CBMPs (Black *et al*., 2019), despite over 60% of UK patients being treated with CBMPs receiving a full-spectrum dried flower product (Olsson *et al*., 2023). Whilst some studies have shown a relationship between recreational cannabis use and anxiety, this evidence is not strong, and some systematic review and meta-analyses even find no association between recreational cannabis use and anxiety (Crippa *et al*., 2009; Gobbi *et al*., 2019).

There is a complete absence of randomized controlled trials which have sought to evaluate the long-term effects of CBMPs in individuals with an anxiety disorder. Consequently, there has been a reliance on observational studies to advance current knowledge and clinical practice, whilst randomized controlled trials are awaited (Banerjee *et al*.,2022). The UK Medical Cannabis Registry was established in 2019 and is the largest CBMP-specific patient registry in the UK, with data published on autism spectrum disorder, post-traumatic stress disorder, depression, and attention-deficit/hyperactivity disorder, alongside chronic physical health conditions (Erridge *et al*., 2021, 2022, 2023a, 2023b; Kawka *et al*., 2021; Ergisi *et al*., 2022, 2023; Harris *et al*., 2022; Mangoo *et al*., 2022; Nimalan *et al*.,2022; Pillai *et al*., 2022; Bapir *et al*., 2023; Dalavaye *et al*., 2023; Ittiphakorn *et al*., 2023; Nicholas *et al*., 2023; Olsson *et al*., 2023; Rifkin-Zybutz *et al*., 2023; Tait *et al*., 2023b; Wang *et al*., 2023). Across most of these studies, improvements in generalized anxiety symptoms have been observed as either a primary or secondary outcome of treatment. Project Twenty21, another CBMP patient registry in the UK, similarly reported improved generalized anxiety symptoms in a cohort of patients followed up for three months (Lynskey *et al*., 2023). In the most recent analysis of patients prescribed CBMPs for GAD from the UK Medical Cannabis Registry 38.3% and 43.6% of participants reported a ≥ 50% reduction or minimal clinically important difference in generalized anxiety severity at 6 months (Rifkin-Zybutz *et al*., 2023). However, this data is affected by the heterogeneity of prescribed CBMPs, alongside other implicit biases. Therefore, the primary aim of this study is to analyze key changes in anxiety-specific and general HRQoL outcomes and safety in patients with GAD prescribed a homogenous selection of CBMPs, to reduce the associated biases that otherwise affect UK Medical Cannabis Registry data. Secondary aims are to report the incidence of adverse events during CBMP therapy for GAD.

## Methods

### Study design and participants

The UK Medical Cannabis Registry (UKMCR) is the first prospective registry to collect pseudonymised outcome data on patients prescribed CBMPs in the UK (Erridge *et al*., 2021), enrolling more than 15 000 patients.

Using the UKMCR, a prospective clinical case series on patients prescribed Adven (Curaleaf International Guernsey, UK) CBMPs for GAD was conducted, as these were identified as the most prescribed CBMPs in prior analyses (Olsson *et al*., 2023; Rifkin-Zybutz *et al*., 2023). The UKMCR has obtained ethical approval from the Central Bristol Research Ethics Committee (reference 22/SW/0145). Every patient provided formal, written consent prior to enrollment. Participants were enrolled consecutively. This study was reported in accordance with the Strengthening the Reporting of Observational Studies in Epidemiology guidelines (Vandenbroucke *et al*., 2007).

On initial consultation, a specialist clinician designated a primary indication for CBMP therapy. Patients were prescribed dried flower (Adven, Curaleaf International Guernsey, UK), medium-chain triglyceride oils (Adven, Curaleaf International, Guernsey, UK) or a combination of both. The medication prescribed was dictated by a consultant physician according primarily to individual patient characteristics incorporating patient preferences. Recording of CBMP prescription data included formulation, daily THC and CBD doses (mg/day) and strain.

Patients on the UKMCR who were enrolled for a minimum of 12 months on the UKMCR with a primary diagnosis of GAD and were only prescribed Adven CBMPs (Curaleaf International Guernsey, UK) were included in this study. Patients continued with pre-existing treatments for anxiety and any changes were recorded. Individuals who did not complete baseline patient-reported outcome measures (PROMs) were excluded from analysis.

### Data collection

Baseline data collection included demographic data: age, sex, occupation, and BMI. Weekly alcohol consumption, tobacco use history, and prior cannabis usage were also recorded. Cannabis usage was quantified using ‘cannabis gram years’, a metric calculated by multiplying mean daily cannabis consumption (grams) by years of prior cannabis use (Erridge *et al*., 2021).

At initial assessment, clinicians also recorded comorbidity data. Comorbidity data was used to later calculate each patient’s Charlson comorbidity index, used to predict 10-year mortality of patients (Charlson *et al*., 2022).

Changes in PROMs were recorded by prompting participants to complete relevant surveys electronically at baseline and 1, 3, 6, and 12 months following CBMP therapy initiation. This method has been evaluated within a patient and public evaluation, which found the platform to be easy to use by most participants (Tait *et al*., 2023a). PROMs included in this study were the GAD-7, the Single-Item Sleep Quality Scale (SQS), EQ-5D-5L, and Patient Global Impression of Change (PGIC). GAD-7, SQS, EQ-5D-5L, and PGIC are all validated and reliable measurement tools for GAD, sleep quality, health-related quality of life (HRQoL), and patients’ perception of change, respectively (Löwe *et al*., 2008; Rampakakis *et al*., 2015; Snyder *et al*., 2018; Feng *et al*., 2021).

### Generalized anxiety disorder-7

The GAD-7 is a seven-item tool that assesses severity of GAD. Each item describes a symptom of GAD and subjects are asked to choose which of the following four categories is most accurate to their experience: ‘not at all’, ‘several days’, ‘more than half the days,’ and ‘nearly every day’. Each option is scored 0, 1, 2, and 3 respectively and a total score out of 21 is calculated. Total scores greater than or equal to 5, 10, and 15 correspond to mild, moderate, and severe anxiety, respectively (Löwe *et al*., 2008). A minimal clinically important difference (MCID) is determined as a reduction of 4 points or more (Toussaint *et al*., 2020). The Cronbach’s alpha and intraclass correlation are 0.92 and 0.83, respectively, indicating good internal consistency and test-retest reliability (Spitzer *et al*., 2006).

### Single-Item Sleep Quality Scale

The SQS is a single-item questionnaire in which subjects rate the quality of their last seven days of sleep on a scale of 0–10. A score of 0 is ‘terrible’, 1–3 ‘poor’, 4–6 ‘fair’, 7–9 ‘good’ and 10 ‘excellent’ (Snyder *et al*., 2018). A score of 3 or less is determined as sleep impaired (Snyder *et al*., 2018). The SQS has moderate test-retest reliability in patients with insomnia (Snyder *et al*., 2018).

### EQ-5D-5L

The EQ-5D-5L consists of five domains: mobility, self-care, usual activities, pain/discomfort, and anxiety/depression. Subjects score each on a five-level scale from ‘no problems’ to ‘extreme problems’ which corresponds to a score of 1–5 (Herdman *et al*., 2011). These scores are used to generate a five-digit code which is mapped to an EQ-5D-5L index value valid for the UK population. The methodology used is detailed by Van Hout *et al.* (2012), the preferred method according to the NICE guidelines (NICE, 2019). An index value of 0 represents a HRQoL worse than death and an index value of 1 represents optimal HRQoL. The test-retest reliability of the index value has been consistently shown to be good (intraclass correlation ≥0.70) across multiple settings.

### Patient global impression of change

The PGIC is a single-item questionnaire which asks patients to complete this statement: ‘Since beginning treatment at this clinic, how would you describe the change (if any) in activity limitations, symptoms, emotions, and overall quality of life-related to your condition?’ Patients select an answer on a scale of 1–7 corresponding where 1 represents ‘no change (or condition has got worse)’ and 7 represents ‘a great deal better, and a considerable improvement that has made all the difference’ (Rampakakis *et al*., 2015).

### Missing data

On graphical assessment, data was adjudged to be missing not at random. To account for missing data, a baseline observation carried forward (BOCF) approach was utilized to provide a more conservative estimation of outcomes in the event of loss to follow-up (Haukoos and Newgard, 2007).

### Adverse events

Adverse events were recorded via an online reporting platform (Tait *et al*., 2023a). Participants have the facility to log into the reporting platform and record an adverse event contemporaneously when an adverse event is experienced. Following the completion of each round of PROMs patients are also directed to the same form to report adverse events if appropriate. These are completed in free text to allow reporting in lay language, which is mapped to appropriate terminology (National Cancer Institute, 2021). Finally, if still unreported, clinicians could record adverse events through a clinician reporting portal during routine follow-up. Adverse events were graded in accordance with the Common Terminology Criteria for Adverse Events version 4.0 as ‘Mild’, ‘Moderate’, ‘Severe’, and ‘Life-threatening’ (National Cancer Institute, 2021).

### Statistical analysis

Data relating to demographics, comorbidities, CBMP prescriptions, and adverse events was analyzed using descriptive statistics. Where appropriate, data was presented as mean (±SD), median (interquartile range) or frequency (%).

Statistical differences in PROMs data from baseline were analyzed using a repeated measures one-way analysis of variance (ANOVA) tests using the Greenhouse-Geiser correction. A post-hoc pairwise comparison was conducted for those variables with statistically significant findings on repeated measures ANOVA, to which Bonferroni correction was applied. A univariate and multivariate analysis was performed to identify variables with increased odds of clinically significant reductions in GAD-7. Statistical significance was defined as *P*-value<0.050.

## Results

**Fig. 1:**
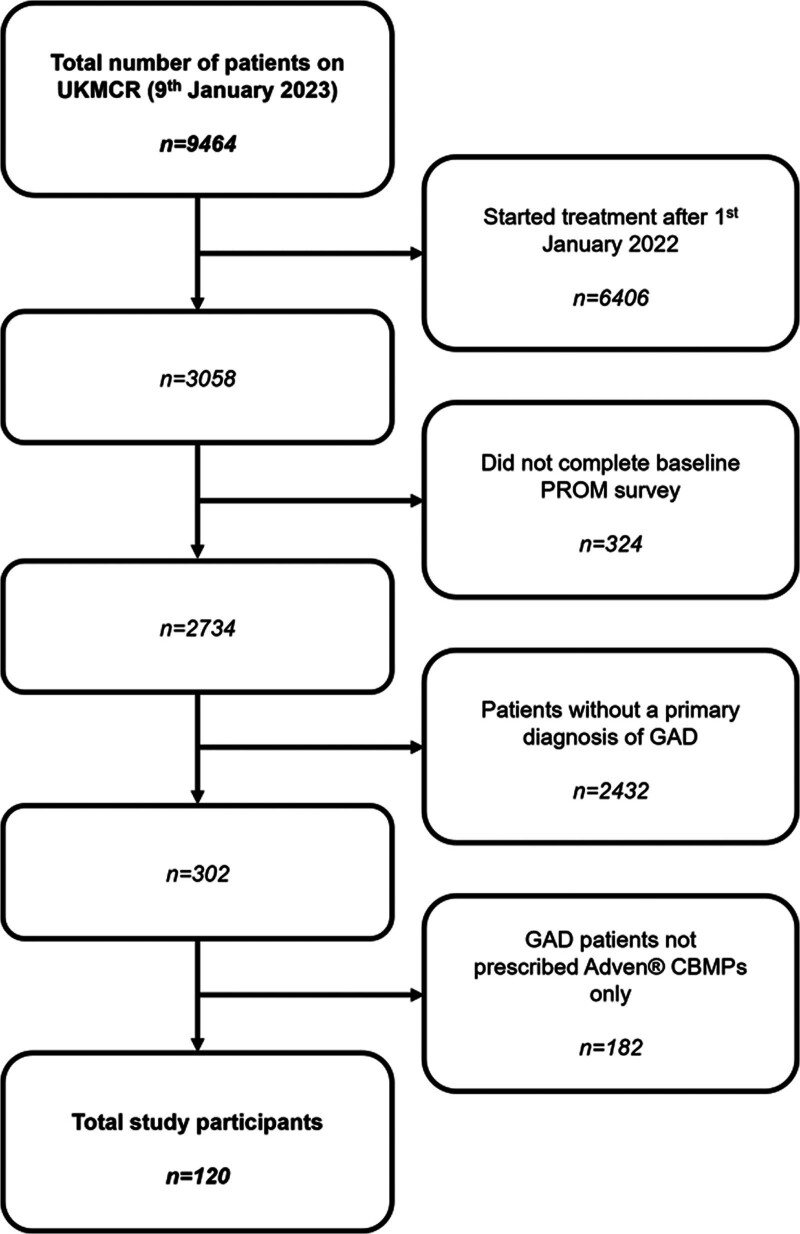
Flowchart detailing exclusion criteria and n numbers for each criterion. CBMPs, Cannabis-Based Medicinal Products; GAD, Generalized Anxiety Disorder; UKMCR, UK Medical Cannabis Registry.

From 9464 patients’ data extracted from the UKMCR, 120 patients were included for final analysis (Fig. 1).

### Demographic data

Participant demographic data for study participants was analyzed (Table 1). The mean age was 39.38 (±12.48) years. Seventy-four (61.67%) participants were male and 46 (38.33%) participants were female. Mean BMI was 27.80 (±12.48) kg/m^2^. The most frequently recorded occupation was ‘unemployed’ (n = 28, 23.33%). There was no statistically significant difference between any demographic variables between those individuals with and without missing PROM data (*P* > 0.050).

**Table 1:** Demographic data and tobacco, alcohol, and cannabis history collected at baseline (n = 120)

Demographic category	n (%)/mean ± SD
Sex	
Male	74 (61.67%)
Female	46 (38.33%)
Age (years)	39.38 ± 12.48
BMI (kg/m^2^)	27.80 ± 8.25
Underweight	3 (2.50%)
Healthy	41 (34.17%)
Overweight	37 (30.83%)
Obese	28 (23.33%)
Unknown	11 (9.17%)
Occupation	
Clerical support workers	8 (6.67%)
Craft and related trades workers	9 (7.50%)
Elementary occupation	5 (4.17%)
Managers	8 (6.67%)
Professional	15 (12.5%)
Service and sales workers	8 (6.67%)
Skilled agricultural, forestry, and fishery workers	1 (0.83%)
Technicians and associate professionals	5 (4.17%)
Unemployed	28 (23.33%)
Other occupations	18 (15.00%)
Unknown	15 (12.50%)

Smoking, alcohol, and cannabis history	n (%)/median (IQR)
Smoking status	
Current smoker	45 (37.50%)
Ex-smoker	47 (39.17%)
Never smoked	28 (23.33%)
Smoking pack years (current and ex-smokers)	6.50 (3.00–19.50)
Weekly alcohol consumption (units)	0.00 (0.00–6.75)
Recreational cannabis usage status	
Current user	59 (49.17%)
Ex-user	31 (25.83%)
Never used	30 (25.00%)
Cannabis gram years (current and ex-users)	5.00 (1.00–15.00)

### Tobacco, alcohol, and cannabis history data

Participant tobacco, alcohol, and cannabis history data were analyzed (Table 1). Ninety-two (76.67%) participants were current or ex-smokers with a median pack year history of 6.50 (3.00–19.50). Median weekly alcohol consumption was 0.00 (0.00–6.75) units. Ninety (75.00%) participants were current or ex-users of cannabis (n = 59, 49.2% and n = 31, 25.8% respectively). Median lifetime cannabis consumption for current and ex-users was 5.00 (1.00–15.00) grams per year. There was no difference in prior tobacco, alcohol, and cannabis use between those individuals with and without missing PROM data (*P* > 0.050).

### Indications for CBMP prescription data

Indications for CBMP prescription were analyzed (see Table A, Supplemental digital content 1, http://links.lww.com/ICP/A128). As per inclusion criteria, all (100%) participants had a primary indication for treatment with CBMPs of GAD. The most common secondary indication was depression (n = 24, 20.00%) and the most common tertiary indications were depression and insomnia (both n = 4, 3.33%).

### Comorbidity data

Participant comorbidity data was analyzed (see Table B, Supplemental digital content 1, http://links.lww.com/ICP/A128). Median Charlson Comorbidity Index score was 0.00 (0.00–1.00).

### CBMP prescription data

Thirty-eight (31.67%) patients were prescribed oil-based CBMPs, 52 (43.33%) were prescribed dried flowers, and 30 (25.00%) were prescribed both. Most participants were prescribed a combination of CBD and THC (n = 104, 86.67%), 15 (12.50%) participants were prescribed THC only, and one (0.83%) participant was prescribed CBD only. Median CBD dose was 31.25 (5.00–55.00) mg and median THC dose was 100.00 (10.00–150.00) mg. There was no difference in CBD or THC dose in those individuals with and without missing PROM data (*P* > 0.050). The most prescribed dried flos was Adven EMT1 (Curaleaf International, UK). The most common medium-chain triglyceride oils were Adven 50 mg/ml CBD (Curaleaf International, UK) and Adven 20 mg/ml THC (Curaleaf International, UK).

### Missing PROMs data

As per inclusion criteria, 100.00% (n = 120) of participants completed every PROM at baseline. At 1-month follow-up twenty-one (17.50%) participants failed to complete the GAD-7, SQS, and EQ-5D-5L. At 3 months of follow-up 35 (29.17%) participants, at 6 months of follow-up 50 (41.67%) participants and at 12 months of follow-up, 71 (59.17%) participants failed to complete the GAD-7, SQS, and EQ-5D-5L. As per the study protocol this data was treated with a BOCF method.

### PROMs analysis

Analysis of longitudinal changes in HRQoL are presented in Table 2. There were improvements in GAD-7 and SQS at 1, 3, 6, and 12 months (*P* < 0.001). There were also improvements in EQ-5D-5L index value at 1, 3, 6 (*P* < 0.001), and 12 months (*P* = 0.006). Regarding the EQ-5D-5L domains, there were statistically significant improvements in usual activities at 1 month (*P* = 0.039) and 3 months (*P* = 0.007); pain and discomfort at 1 (*P* = 0.001), 3 (*P* = 0.015), and 6 months (*P* = 0.002) and anxiety and depression at 1, 3, 6 (*P* < 0.001) and 12 months (*P* = 0.007).

**Table 2:** Mean baseline and follow-up GAD-7, SQS, and EQ-5D-5L at 1, 3, 6, and 12 months (n = 120)

Patient-reported outcome measure	Follow-up (months)	Mean ± SD	*P*-value compared to previous follow-up period	*P*-value compared to baseline
GAD-7	Baseline	13.87 ± 0.50		
	1	9.51 ± 0.55	<0.001	
	3	9.717 ± 0.57	1.000	<0.001
	6	9.97 ± 0.60	1.000	<0.001
	12	11.12 ± 0.60	0.129	<0.001
SQS	Baseline	3.77 ± 0.21		
	1	5.64 ± 0.23	<0.001	
	3	5.50 ± 0.24	1.000	<0.001
	6	4.99 ± 0.27	0.071	<0.001
	12	4.73 ± 0.26	1.000	<0.001
EQ-5D-5L mobility	Baseline	1.50 ± 0.08		
	1	1.43 ± 0.08	1.000	
	3	1.46 ± 0.08	1.000	1.000
	6	1.52 ± 0.08	1.000	1.000
	12	1.50 ± 0.08	1.000	1.000
EQ-5D-5L self-care	Baseline	1.51 ± 0.09		
	1	1.47 ± 0.09	1.000	
	3	1.45 ± 0.08	1.000	1.000
	6	1.54 ± 0.09	0.337	1.000
	12	1.53 ± 0.09	1.000	1.000
EQ-5D-5L usual activities	Baseline	2.33 ± 0.12		
	1	2.06 ± 0.11	0.039	
	3	1.98 ± 0.11	1.000	0.007
	6	2.15 ± 0.11	0.194	0.399
	12	2.13 ± 0.11	1.000	0.141
EQ-5D-5L pain and discomfort	Baseline	2.13 ± 0.11		
	1	1.83 ± 0.09	0.001	
	3	1.89 ± 0.10	1.000	0.015
	6	1.89 ± 0.10	1.000	0.002
	12	1.98 ± 0.10	0.858	0.074
EQ-5D-5L anxiety and depression	Baseline	3.43 ± 0.10		
	1	2.89 ± 0.10	<0.001	
	3	2.94 ± 0.11	1.000	<0.001
	6	2.94 ± 0.11	1.000	<0.001
	12	3.15 ± 0.10	0.180	0.007
EQ-5D-5L index value	Baseline	0.53 ± 0.03		
	1	0.64 ± 0.03	<0.001	
	3	0.63 ± 0.03	1.000	<0.001
	6	0.61 ± 0.03	1.000	<0.001
	12	0.58 ± 0.03	0.848	0.006
PGIC	1	5.29 ± 0.17		
	3	5.55 ± 0.13	0.223	-
	6	5.42 ± 0.16	1.000	-
	12	5.59 ± 0.15	1.000	-

PGIC data was collected at 1, 3, 6, and 12 months. Twenty-three (19.17%) participants had missing PGIC scores at 1 month and 12 months. Twenty-one (17.50%) participants had missing PGIC scores at 3 and 6 months. The median PGIC score was 6.00 (5.00–6.00) at each follow-up period. At 12 months follow-up, nine (7.50%) participants reported a PGIC of 1–3 (no noticeable change or worsening of condition), one (0.83%) participant reported a PGIC of 4 (better, but not made any real difference) and 87 (72.50%) reported a PGIC of 5–7 (better, with a noticeable improvement).

There was a clinically significant (4 points or greater) reduction in GAD-7 score for thirty-two (26.67%) participants at 12 months compared to baseline (Table 3). Twenty (32.79%) patients with severe baseline GAD-7 scores had clinically significant reductions in GAD-7 at 12 months. Seven (21.88%) participants with moderate baseline GAD-7 scores had clinically significant reductions in GAD-7 at 12 months. Five (27.78%) participants with mild baseline GAD-7 scores had a clinically significant reduction in GAD-7 score.

**Table 3:** Two-way frequency table showing participants’ GAD-7 classification at baseline and 12 months (n = 120)

		GAD-7 score at 12 months				Total
		0–4 (minimal)	5-9 (mild)	10–14 (moderate)	15–21 (severe)	
GAD-7 score at baseline	0–4 (minimal)	8	1	0	0	9(7.50%)
	5–9 (mild)	5	13	0	0	18(15.00%)
	10–14 (moderate)	4	3	22	3	32(26.67%)
	15–21 (severe)	8	9	3	41	61(50.83%)
	Total	25(20.83%)	26(21.67%)	25(20.83%)	44(36.67%)	120

Univariate logistic regression assessing the association between variables and the likelihood of experiencing a clinically significant benefit in GAD-7 at 12 months (Table C, Supplemental digital content 1, http://links.lww.com/ICP/A128) showed those taking a dried flower CBMP preparation and those with a daily THC dose greater than 100 mg to have increased odds (OR = 3.800, 95%CI = 1.269–11.381, *P* = 0.017 and OR = 2.714, 95%CI = 1.062–6.937, *P* = 0.037 respectively).

Multivariate logistic regression (Table 4) showed those aged 41–50 years old and those with a baseline GAD-7 score of 15–21 (severe) to have increased odds of clinically significant improvement in GAD-7 (OR = 6.721, 95%CI = 1.334–33.858, *P* = 0.021 and OR = 16.018, 95%CI = 2.157–118.693, *P* = 0.007 respectively).

**Table 4:** Multivariate analysis assessing association between variables and the likelihood of experiencing a clinically significant benefit in GAD-7 at 12 months

Variable	n	OR (95% CI)	*P*-value
Age			
<30	28	1	
31–40	41	2.674 (0.621–11.506)	0.187
41–50	23	6.721 (1.334–33.858)	0.021*
51–60	12	1.852 (0.280–12.240)	0.523
60+	5	1.156 (0.057–23.424)	0.925
Gender			
Male	67	1	
Female	42	1.568 (0.467–5.261)	0.466
BMI (kg/m^2^)			
<25	44	1	
25–30	37	0.435 (0.109–1.731)	0.237
30–35	12	1.662 (0.280–9.868)	0.576
>35	16	3.911 (0.845–18.108)	0.081
Prior cannabis usage status			
Current user	53	1	
Ex-user	29	1.031 (0.299–3.551)	0.961
Never used	27	0.958 (0.196–4.685)	0.958
CBMP prescription			
Oils	34	1	
Dried flower	48	21.964 (0.752–641.491)	0.073
Both	27	11.563 (0.442–302.527)	0.142
CBD contents in CBMP			
No CBD	13	1	
≤ Median dose of cohort (≤31.25 mg/day)	41	4.583 (0.625–33.605)	0.134
> Median dose of cohort (>31.25 mg/day)	55	3.248 (0.417–25.316)	0.261
THC contents in CBMP			
≤ Median dose of cohort (≤100.00 mg/day)	40	1	
> Median dose of cohort (>100.00 mg/day)	69	0.311 (0.021–4.680)	0.398
Baseline GAD-7 score			
0–9 (minimal or mild)	23	1	
10–14 (moderate)	30	4.537 (0.735–28.004)	0.103
15–21 (severe)	56	16.018 (2.157–118.963)	0.007**
Baseline SQS score			
≤ 3 (sleep impaired)	61	1	
> 3 (non-sleep impaired)	48	0.991 (0.320–3.066)	0.987

Significant differences are denoted by asterisks (*P < 0.050,**P = 0.010, and ***P < 0.001).

### Changes in medication

Seventy-seven (64.17%) of the cohort were being treated with antidepressants. Sixty-two (80.52%) patients had no change in their antidepressant medication over the 12 month period, four (5.19%) reduced dose, six (7.79%) stopped completely, one (1.30%) patients increased dose, and four (5.19%) started a new antidepressant (Table D, Supplemental digital content 1, http://links.lww.com/ICP/A128). Twenty-four (20.00%) patients were being treated with benzodiazepines. Nineteen (79.17%) patients had no change in benzodiazepine medication, one (4.17%) reduced dose, three (12.50%) stopped completely and one (4.17%) patient started a new benzodiazepine (Table D, Supplemental digital content 1, http://links.lww.com/ICP/A128). Nine (7.50%) patients were being treated with insomnia-related medications. Seven (77.78%) patients had no change in insomnia-related medication and two (22.22%) patients changed to a new insomnia-related medication (Table D, Supplemental digital content 1, http://links.lww.com/ICP/A128).

### Adverse event data

Participants reported adverse events were analyzed (Table 5). Twenty-four (20.00%) patients reported a total of 442 (368.33%) adverse events. The most common adverse events were concentration impairment and dry mouth (both n = 35, 7.92%). One hundred and eighty-four (41.63%) adverse events reported were mild, 44.57% (n = 197) were moderate and 13.80% (n = 61) were severe. There were no life-threatening or disabling adverse event reports. Adverse event data was not suitable for logistic regression analysis due to limitations of sample size.

**Table 5:** Adverse events reported by participants (n = 120)

Adverse event	Mild	Moderate	Severe	Total (%)
Abdominal pain	3	2	0	5 (1.13%)
Agitation	0	1	0	1 (0.23%)
Akathisia	0	1	0	1 (0.23%)
Amnesia	4	16	6	26 (5.88%)
Anorexia	2	3	2	7 (1.58%)
Anxiety	3	4	3	10 (2.26%)
Ataxia	5	5	0	10 (2.26%)
Blurred vision	11	1	0	12 (2.71%)
Bruxism	1	0	0	1 (0.23%)
Chest pain	1	0	0	1 (0.23%)
Cognitive disturbance	6	15	3	24 (5.43%)
Concentration impairment	14	19	2	35 (7.92%)
Confusion	7	3	2	12 (2.71%)
Constipation	5	1	0	6 (1.36%)
Costochondritis	1	0	0	1 (0.23%)
Delirium	4	3	1	8 (1.81%)
Depression	1	4	15	20 (4.52%)
Dissociation	0	2	0	2 (0.45%)
Dizziness	12	6	1	19 (4.30%)
Dry mouth	20	15	0	35 (7.92%)
Dysgeusia	7	5	2	14 (3.17%)
Dyspepsia	4	0	1	5 (1.13%)
Fall	1	0	0	1 (0.23%)
Fatigue	8	11	3	22 (4.98%)
Fever	1	0	0	1 (0.23%)
Generalized muscle weakness	0	5	1	6 (1.36%)
Headache	10	4	3	17 (3.85%)
Hypertension	1	0	0	1 (0.23%)
Insomnia	4	9	5	18 (4.07%)
Lethargy	11	13	0	24 (5.43%)
Libido decreased	1	0	0	1 (0.23%)
Nausea	14	0	2	16 (3.62%)
Palpitations	0	1	0	1 (0.23%)
Paranoia	2	5	0	7 (1.58%)
Pharyngitis	0	5	0	5 (1.13%)
Rash	0	4	0	4 (0.90%)
Seizure	0	0	2	2 (0.45%)
Sensory overload	0	0	1	1 (0.23%)
Sinus pain	1	0	0	1 (0.23%)
Sneezing	1	0	0	1 (0.23%)
Somnolence	0	25	6	31 (7.01%)
Toothache	0	1	0	1 (0.23%)
Tremor	3	1	0	4 (0.90%)
Upper respiratory infection	0	2	0	2 (0.45%)
Urinary tract infection	0	4	0	4 (0.90%)
Vertigo	6	1	0	7 (1.58%)
Vomiting	3	0	0	3 (0.38%)
Weight loss	6	0	0	6 (1.36%)
Total	184 (41.6%)	197 (44.6%)	61 (13.8%)	442 (368.33%)

## Discussion

This study analyzed outcomes in GAD patients enrolled in the UKMCR. Improvements in GAD-7, SQS, EQ-5D-5L index, and EQ-5D-5L anxiety and depression scores up until 12 months follow-up demonstrate an association between CBMP treatment initiation and improvements in HRQoL measures in GAD patients. Additionally, one-quarter of patients reported a minimal clinically important difference in anxiety at 12 months. Only twenty-four (20.00%) patients reported adverse events, the majority of which were mild or moderate, suggesting that CBMPs were largely well-tolerated by participants in this study. However, these outcomes must be appreciated within the context of the limitations of the study design.

Improvements in GAD-7 score were observed at all follow-up periods in this study, with one-quarter reporting clinically significant reductions at 12 months. Stith *et al*. previously reported an association between self-directed cannabis flower use and improvements in anxiety severity (Stith *et al*., 2020). Whilst this corroborates the findings of this study, anxiolytic effects were not investigated longitudinally with symptoms monitored up to 4 h post-CBMP administration. When considering these findings with the findings of this study, a more complete understanding of CBMP therapy’s association with anxiety improvement, in the short-term and long-term, may be reached. Multivariate analysis of this study showed an association between patients on CBMPs with severe baseline GAD-7 scores and clinically significant improvements in GAD-7 compared to moderate, mild, and subclinical GAD-7 CBMP patients (*P* = 0.007). A 2021 review concluded that greater anxiety symptoms are linked with a poorer quality of life (Wilmer *et al*., 2021). Consequently, the findings of this study provide promise for a treatment which is potentially more effective for patients with severe anxiety. This is important considering 50% and 30% of patients with GAD will not respond to first-line therapies and multiple medications, respectively.

Anxiety was also reported as an adverse event during the present study, with an incidence of 2.26%. Although the adverse events are not assessed to determine if they are treatment-related, the potential anxiogenic effects of THC must also be considered (Rey *et al*., 2012; Stoner, 2017). This is particularly important, considering the median daily THC dose was 100.00 mg/day, which is high relative to studies which have evaluated anxiety as an outcome in the past (Bystritsky, 2006; Ansara, 2020; Black *et al*., 2019).

This study suggests an association between improvements in sleep quality and CBMP therapy. A 2018 study investigating cannabis flower’s effect on insomnia reported improvements in perceived insomnia (Vigil *et al*., 2018). Considering sleep disorders are one of the most common reasons cited for CBMP use (Hazekamp *et al*., 2013), these findings are particularly promising for future research. This study also reported that CBMP patients with baseline sleep impairment were not more likely to report clinically significant improvements in GAD-7 compared to patients with no sleep impairment at baseline on multivariate logistic regression. This suggests that sleep quality was not a confounding factor for improvement in GAD-7 in this cohort.

Observed improvements in HRQoL are supported by a previous clinical case series of GAD patients enrolled on the UKMCR. Statistically significant improvements in EQ-5D-5L were associated with CBMP therapy in GAD patients, concordant with the findings of this study (Ergisi *et al*., 2022). This illustrates a potentially reproducible associated improvement in HRQoL with CBMP therapy for GAD patients. This study adds to the limited evidence regarding CBMP therapy’s effect on HRQoL, suggesting an association between the two in GAD patients. However, further research would need to be conducted to further investigating CBMP’s effects on HRQoL in a broader patient population.

CBMP therapy appears to be tolerated well by the majority of this cohort. Due to the design of this study, adverse events due to CBMP therapy were unable to be distinguished from adverse events resulting from natural or coincidental causes. For example, the most frequently reported severe adverse event was depression, which GAD is a significant risk factor for (Hirschfeld, 2001). As a result, adverse event data should be interpreted with caution. Additionally, drug-drug interactions were not investigated as a part of this study, which also may potentially exacerbate adverse events. Despite this, these findings suggest CBMPs are well-tolerated in this population.

### Limitations

Due to the observational nature of this study, no causative relationship can be established between CBMP therapy and improvements in GAD, sleep, and HRQoL outcomes. Internal validity was limited because of a lack of blinding and randomization. With no control arm to this trial, genuine CBMP treatment effects cannot be distinguished from potential confounding effects, such as regression to the mean. Another example was that many patients were former or current tobacco smokers, which is known to be associated with increased anxiety symptoms (Moylan *et al*., 2013). In addition, despite PROMs being the gold-standard assessment for subjective symptoms of anxiety, they remain subject to recall bias. PROMs are also affected by ceiling effects, which might be responsible for those individuals with the highest GAD-7 scores at baseline being the most likely to report a clinically significant improvement at 12 months. Additionally, the placebo effect of CBMPs may be exaggerated secondary to the expectancy bias shown to be associated through positive media reporting (Gedin *et al*., 2022). Moreover, paying for medications has been shown to increase perceived medication quality (Díaz-Lago *et al*., 2023). CBMPs are also associated with an exaggerated placebo effect due to the associated biological effects and aroma (Gertsch, 2018). Finally, 59 (49.17%) participants had previously acquired cannabis prior to enrollment, which may also lead to expectancy bias. However, these individuals may conversely have developed tolerance to the effects of CBMPs, which would bias the results towards a null finding. Therefore, the positive findings suggest that there may be supplementary benefits to sourcing CBMPs compared to illicit cannabis, such as consistent supply of pharmaceutical quality medication overseen by a specialist physician. Moreover, this may also lead to a reduction in anxiety through reducing engagement in illegal activity. However, it has been shown by our group that medical cannabis patients still perceive themselves to be subject to stigma (Troup *et al*., 2022). This is just one example of the effects of sampling bias, including a higher than anticipated proportion of male participants compared to the general population with GAD. Finally, it was not possible to screen for the development of cannabis use disorder, considering currently available screening tools have at best not been validated for clinical populations and at worse heavily utilize questions around frequency of cannabis use, which are inappropriate for populations who are prescribed CBMPs daily.

### Conclusion

This study reports that initiation of Adven (Curaleaf International, Guernsey, UK) CBMP treatment is associated with significant improvement in HRQoL outcomes in this population of GAD patients. This suggests the therapeutic potential of CBMPs for GAD. Although limitations due to study design mean that a causative relationship cannot be established, these findings suggest that the benefits of CBMPs may be most marked in individuals with severe anxiety at baseline. The findings of this study can act as the basis for future controlled studies, with study designs that can infer causative relationships, and determine the optimal dosing strategies, and most appropriate populations who may benefit from this treatment.

## Acknowledgements

Data that support the findings of this study are available from the UK Medical Cannabis Registry. Restrictions apply to the availability of these data. Data specifications and applications are available from the corresponding author.

All authors have contributed to and approved the final manuscript.

The authors confirm that the PI for this paper is Mikael H. Sodergren and that he had direct clinical responsibility for patients.

Ethical approval provided by Central Bristol Research Ethics Committee (Reference: 22/SW/0145).

Patient consent statement: All participants completed written, informed consent prior to enrollment in the registry.

### Conflicts of interest

Adam Li is a medical student at Imperial College London. Adam Li has no shareholdings in pharmaceutical companies. Simon Erridge is a junior doctor and is the Head of Research at Sapphire Medical Clinics. Simon Erridge is an honorary clinical research fellow at Imperial College London. The views expressed are those of the author(s) and not necessarily those of the NHS. Simon Erridge has no shareholdings in pharmaceutical companies. Carl Holvey is Chief Clinical Pharmacist at Sapphire Medical Clinics. Carl Holvey has no shareholdings in pharmaceutical companies. Ross Coomber is a consultant orthopedic surgeon, Operations Director at Sapphire Medical Clinics and a consultant at St George’s Hospital, London. The views expressed are those of the author(s) and not necessarily those of the NHS. Ross Coomber has no shareholdings in pharmaceutical companies. Daniela Barros is a consultant psychiatrist at Sapphire Medical Clinics. Daniela Barros has no shareholdings in pharmaceutical companies. Urmila Bhoskar is a consultant psychiatrist at Sapphire Medical Clinics. Urmila Bhoskar has no shareholdings in pharmaceutical companies. Mathieu Crews is a consultant psychiatrist at Sapphire Medical Clinics. Mathieu Crews has no shareholdings in pharmaceutical companies. Lorna Donnelly is a consultant psychiatrist at Sapphire Medical Clinics. Lorna Donnelly has no shareholdings in pharmaceutical companies. Muhammad Imran is a consultant psychiatrist at Sapphire Medical Clinics. Muhammad Imran has no shareholdings in pharmaceutical companies. Laura Korb is a consultant psychiatrist at Sapphire Medical Clinics and North London Mental Health Partnership. Laura Korb has no shareholdings in pharmaceutical companies. The views expressed are those of the author(s) and not necessarily those of the NHS. Gracia Mwimba is a consultant psychiatrist at Sapphire Medical Clinics. Gracia Mwimba has no shareholdings in pharmaceutical companies. Simmi Sachdeva-Mohan is a consultant psychiatrist at Sapphire Medical Clinics. Simmi Sachdeva-Mohan has no shareholdings in pharmaceutical companies. James Rucker is a consultant psychiatrist and a former director at Sapphire Medical Clinics (London). James Rucker is an honorary consultant psychiatrist at The South London & Maudsley NHS Foundation Trust, and an NIHR Clinician Scientist Fellow at the Centre for Affective Disorders at King’s College London. James Rucker is funded by a fellowship (CS-2017-17-007) from the National Institute for Health Research (NIHR). The views expressed are those of the author(s) and not necessarily those of the NHS, the NIHR, or the Department of Health. James Rucker has no shareholdings in pharmaceutical companies. James Rucker reviewed this article and made comments. Mikael Sodergren is a consultant hepatopancreatobiliary surgeon at Imperial College NHS Trust. He is the Chief Medical Officer at Curaleaf International. He is a senior clinical lecturer at Imperial College London. The views expressed are those of the author(s) and not necessarily those of the NHS. For the remaining authors, there are no conflicts of interest.

## Supplementary Material


