# Supplementary material for: UK Medical Cannabis Registry: a case series analyzing clinical outcomes of medical cannabis therapy for generalized anxiety disorder patients
Source: Int Clin Psychopharmacol. 2024 Feb 2;39(6):350–60. doi: 10.1097/YIC.0000000000000536 (PMC11424060; doi:10.1097/YIC.0000000000000536)
Supplement: Supplementary file 1 [file icp-39-350-s001.pdf]

Supplemental Digital Content:

Table A. Primary, secondary, and tertiary indications for being prescribed cannabis-based medicinal products (n=120)

**Indication**	**Primary n (%)**	**Secondary n (%)**	**Tertiary n (%)**
Anxiety	120 (100%)	-	-
Agoraphobia	-	2 (1.7%)	1 (0.8%)
Attention deficit hyperactivity disorder	-	3 (2.5%)	0 (0.0%)
Autistic spectrum disorder	-	2 (1.7%)	2 (1.7%)
Cancer pain	-	1 (0.8%)	0 (0.0%)
Chronic pain	-	2 (1.7%)	2 (1.7%)
Depression	-	24 (20.0%)	4 (3.3%)
Eating disorder	-	1 (0.8%)	2 (1.7%)
Fibromyalgia	-	1 (0.8%)	0 (0.0%)
Headache	-	1 (0.8%)	0 (0.0%)
Insomnia	-	7 (5.8%)	4 (3.3%)
Obsessive compulsive disorder	-	2 (1.7%)	0 (0.0%)
Panic disorder	-	1 (0.8%)	0 (0.0%)
Parkinson's disease	-	1 (0.8%)	0 (0.0%)
Post-traumatic stress disorder	-	3 (2.5%)	1 (0.8%)

Table B. Comorbidity frequencies of study participants (n=120)

**Comorbidity**	**n (%)/median (IQR)**
Myocardial infarction	0 (0.0%)
Congestive heart failure	1 (0.8%)
Peripheral vascular disease	0 (0.0%)
Cerebrovascular accident or ischaemic attack	0 (0.0%)
Dementia	1 (0.8%)
Chronic obstructive pulmonary disease	2 (1.7%)
Connective tissue disease	1 (0.8%)
Peptic ulcer disease	2 (1.7%)
Liver disease	1 (0.8%)
Diabetes	2 (1.7%)
Hemiplegia	0 (0.0%)
Moderate to severe chronic kidney disease	3 (2.5%)
Solid tumour	
Localised	3 (2.5%)
Metastatic	1 (0.8%)
Leukaemia	0 (0.0%)
Lymphoma	0 (0.0%)
AIDS	0 (0.0%)
Charlson Comorbidity Index score	0.00 (0.00-1.00)
Hypertension	11 (9.2%)
Arthritis	1 (0.8%)
Epilepsy	2 (1.7%)
Venous thromboembolism	1 (0.8%)
Endocrine/thyroid dysfunction	4 (3.3%)

Table C. Univariate analysis assessing association between variables and the likelihood of experiencing a clinically significant benefit in GAD-7 at 12 months. Significant differences are denoted by asterisks (*: p<0.050,** p,0.010 and ***: p<0.001)

Variable	n	OR (95% CI)	p-value
Age			
<30	30	1	
31-40	46	1.033 (0.349-3.053)	0.954
41-50	24	2.347 (0.727-7.579)	0.154
51-60	12	1.095 (0.231-5.195)	0.909
60+	8	0.469 (0.049-4.497)	0.512
Gender			
Male	74	1	
Female	46	0.793 (0.341-1.847)	0.793
Body Mass Index (kg/m^2)			
<25	44	1	
25-30	37	0.964 (0.350-2.660)	0.944
30-35	12	1.500 (0.377-5.966)	0.565
>35	16	2.333 (0.702-7.751)	0.167
Prior Cannabis Usage Status			
Current user	59	1	
Ex-user	31	1.330 (0.481-3.675)	0.582
Never used	30	1.143 (0.356-3.673)	0.823
CBMP Prescription			
Oils	38	1	
Dried flower	52	3.800 (1.269-11.381)	0.017*
Both	30	2.400 (0.694-8.300)	0.167
CBD Contents in CBMP			
No CBD	15	1	
≤ Median dose of cohort (≤31.25mg/day)	45	4.333 (0.872-21.543)	0.073
> Median dose of cohort (>31.25mg/day)	60	1.625 (0.332-8.191)	0.556
THC Contents in CBMP			
≤ Median dose of cohort (≤100.00mg/day)	45	1	
> Median dose of cohort (>100.00mg/day)	75	2.714 (1.062-6.937)	0.037*
Baseline GAD-7 score			
0-9 (minimal or mild)	27	1	
10-14 (moderate)	32	1.610 (0.416-6.227)	0.490
15-21 (severe)	61	3.019 (0.922-9.882)	0.068
Baseline SQS score			
≤ 3 (sleep impaired)	69	1	
> 3 (non-sleep impaired)	51	0.900 (0.396-2.048)	0.802

Table D. Frequency of changes in prescription of antidepressants, benzodiazepines and insomnia-related medication

Medication	Total	No Change	Stopped Taking	Reduced Dose	Increased dose	New Medication
Antidepressants	77	62 (80.52%)	6 (7.79%)	4 (5.19%)	1 (1.30%)	4 (5.19%)
Benzodiazepines	24	19 (79.17%)	3 (12.50%)	1 (4.17%)	0 (0.00%)	1 (4.17%)
Insomnia-related	9	7 (77.78%)	0 (0.00%)	0 (0.00%)	0 (0.00%)	2 (22.22%)
